# Peer Review of “Insider Threats to the Military Health System: A Systematic Background Check of TRICARE West Providers”

**DOI:** 10.2196/57159

**Published:** 2024-04-09

**Authors:** Anonymous

**Keywords:** TRICARE, health care fraud, Defense Health Agency, fraud, fraudulent, insurance, coverage, beneficiary, beneficiaries, background check, background checks, demographic, security clearance, FDA, Medicaid, Medicare, provider, provider referral, military, False Claims Act, HIPAA breach, OIG-LEIE, inspector general, misconduct, insider threat, information system, zero trust, data management, Food and Drug Administration, Health Insurance Portability and Accountability Act breach, Office of Inspector General's List of Excluded Individuals and Entities

*This is the peer-review report for “Insider Threats to the Military Health System: A Systematic Background Check on TRICARE West Providers.”*

## Round 1 Review

### General Comments

This paper [1] examines the list of TRICARE providers eligible to deliver telehealth whose names appear on one or more federal sanction lists. This work could have a high impact with implications for national security and patient safety. However, it is not well organized and does not seem to adhere to the scientific method.

### Specific Comments

#### Major Comments

1. This is important work, but is it actually science? A team compared two lists. There are no statistics, minimal numbers, and only one hard conclusion (improper monies). Lots of speculation, but no real answers. Because you are calling out the Defense Health Agency (DHA) and the Military Health System (MHS) on inadequate oversight, your conclusions must be driven by airtight methodology and presented in a professional and well-organized manner. Otherwise, you may just submit this work to the DHA’s Office of Inspector General as you’ve already done and call it completed.
2. Assuming you decide to go with the science, this article has important things to say, but it is not yet ready for publication. It is poorly organized, somewhat informal in tone, and comes across as inflammatory in places. An example of poor organization is the focus on cyber threats and potential in the Introduction, improper monies paid to sanctioned providers in the Results, and a distrust of provider data in the Discussion. Patient safety is not discussed until page 17. Recommend mentioning all these issues in the Introduction and then addressing them in the Results/Discussion in a systematic, organized fashion. Also, streamline areas where the same data is repeated multiple times.
3. Similarly, I recommend keeping all the DHA/MHS recommendations together and at the end of the Discussion section, and addressing these in a systematic and organized fashion. “Based on these results, the DHA/MHS/ TRICARE/ whoever should consider the following: (1) Recommendation 1. (2) Recommendation 2...” etc. (Don’t need to take my wording but this is a general idea.)
4. If there are other people who participated in this study, they should be included as authors or in the Acknowledgments. I doubt that one person compared tens of thousands of names solo.

#### Minor Comments

Everything else is gravy until these major issues are fixed. That said, I sincerely wish you the best of luck as you continue working on this important issue.

## Abbreviations

DHA: Defense Health Agency
MHS: Military Health System
